# Structural basis for the dimerization of Nab2 generated by RNA binding provides insight into its contribution to both poly(A) tail length determination and transcript compaction in *Saccharomyces cerevisiae*

**DOI:** 10.1093/nar/gkw1224

**Published:** 2016-12-08

**Authors:** Shintaro Aibara, James M. B. Gordon, Anja S. Riesterer, Stephen H. McLaughlin, Murray Stewart

**Affiliations:** MRC Laboratory of Molecular Biology, Francis Crick Avenue, Cambridge Biomedical Campus, Cambridge CB2 0QH, UK

## Abstract

In *Saccharomyces cerevisiae* generation of export-competent mRNPs terminates the nuclear phase of the gene expression pathway and facilitates transport to the cytoplasm for translation. Nab2 functions in this process to control both mRNP compaction that facilitates movement through nuclear pore complexes and the length of transcript poly(A) tails. Nab2 has a modular structure that includes seven CCCH Zn fingers that bind to A-rich RNAs and fingers 5–7 are critical for these functions. Here, we demonstrate, using both biophysical and structural methods, that binding A_11_G RNA induces dimerization of Zn fingers 5–7 mediated by the novel spatial arrangement of the fingers promoting each RNA chain binding two protein chains. The dimerization of Nab2 induced by RNA binding provides a basis for understanding its function in both poly(A) tail length regulation and in the compaction of mature transcripts to facilitate nuclear export.

## INTRODUCTION

Nascent transcripts undergo a co-ordinated series of modifications (including 5΄-capping, splicing, and 3΄-cleavage/polyadenylation) in the nucleus to generate mature mRNAs. The generation of export-competent mRNPs (messenger ribonucleoprotein complexes) represents the final step in the nuclear portion of the gene expression pathway and facilitates the transport of mature transcripts through nuclear pore complexes (NPCs) to the cytoplasm for translation. During nuclear processing a broad spectrum of proteins bind to the transcripts. Although some facilitate particular functions and are generally then released, other RNA-binding proteins function to compact mature transcripts to facilitate movement through NPCs (reviewed by (1)). In *Saccharomyces cerevisiae*, the nuclear export of mature transcripts is mediated primarily by the export factor Mex67:Mtr2 that binds to the mRNPs and, through interactions with NPC proteins that contain multiple phenylalanine–glycine motifs (FG-nucleoporins), facilitates their passage though the pores (reviewed by (2–4)). Although mRNP composition probably varies for different transcripts, several mRNA binding proteins, including cap-binding proteins, the export factor, Mex67:Mtr2, the SR protein, Npl3, and Nab2, are thought to be present on most, if not all, export-competent mRNPs in *S. cerevisiae* (5), albeit metazoan mRNPs appear to also have some additional RNA-binding proteins, such as those of the exon-junction complex.

The heterogeneous nuclear ribonuclearprotein, Nab2, a conserved Zn finger protein, is essential for mRNA production (6) and functions in polyadenylation, surveillance and the packaging leading to generation of export-competent mRNPs (reviewed by (7)). *nab2* mutants frequently generate hyperadenylation, defects in surveillance, and reduced mRNA nuclear export resulting in nuclear accumulation of poly(A)-mRNA, but the impact on each of these functions often varies between mutants, consistent with the Nab2 protein having several functions in the gene expression pathway (reviewed by (7)). Nab2 appears to associate with most mRNAs before they are exported (8,9) and, although localized to the nucleus at steady-state, it shuttles between nucleus and cytoplasm (10,11). Nab2 appears to become attached to the mRNP after splicing and during or immediately after polyadenylation and influences the packaging required to generate export-competent mRNPs (8,9).

**Figure 1.**
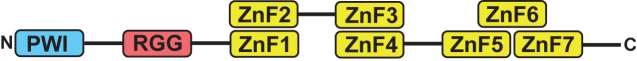
Schematic illustration of the domain structure of *S. cerevisiae* Nab2. The protein is based an N-terminal domain (40), an RGG domain, and seven Zn fingers, joined by flexible linkers. The Zn fingers are clustered into three groups (1+2, 3+4 and 567) and in each group the fingers have defined orientations to one another (17,18).

The *S. cerevisiae* Nab2 protein contains four domains (Figure 1): an N-terminal PWI-like domain that interacts with NPCs, followed by a Gln-rich linker; an Arg-Gly (RGG) domain required for nuclear import; and finally a domain containing seven tandem CCCH Zn fingers that binds polyadenosine-RNA *in vitro*, contributes to poly(A) tail length control, and also functions as a checkpoint for proper 3΄ processing (reviewed by (7)). The Zn finger domain is essential for Nab2 function and for its binding to poly(A) mRNA, albeit only fingers 5–7 are necessary and are sufficient for high affinity polyadenosine–RNA binding (12). Although Nab2 clearly binds to polyadenosine and mRNA poly(A) tails, this protein also binds to other regions of transcripts and is proposed to contribute to the mRNP organization and compaction associated with the generation of export-competent mature transcripts (8). Thus, *S. cerevisiae* mRNPs isolated by TAP-tagged Nab2 pull-downs contain approximately nine Nab2 molecules per kb (8), whereas poly(A) tails probably only bind two or three Nab2s (13). Transcriptome-wide analysis of RNP composition (9) and ChIP (14) also indicate that Nab2 is bound throughout the body of mRNAs in addition to being concentrated at their poly(A) tail. Although Nab2 exhibits high affinity for poly (A) RNA, both SELEX (15) and computational analysis (16) have identified RNA sequence motifs that recognize Nab2 but which, in addition to being A-rich, also contain a 3΄ G.

Solution structures of Zn fingers 1–4 (17) and 5–7 (18) and a crystal structure of the analogous fingers 3–5 of *Chaetomium thermophilum* Nab2 bound to short (2–3 nt) adenosine oligomers (19) all show defined spatial arrangements between the fingers. Thus, in *S. cerevisiae* Nab2, fingers 1–2, 3–4 and 5–7 are grouped into separate units in which the fingers take up distinctive spatial arrangements and a similar grouping was observed in *C. thermophilum* fingers 3–5. Mutations in *S. cerevisiae* Nab2 that disrupt the structure of the fingers, such as C437S (20), or that target residues likely to be involved in RNA binding (18,19) generate longer poly(A) tails *in vivo*.

Here, we demonstrate that binding either A_12_ RNA or the A_11_G RNA motif identified by SELEX (15) to *S. cerevisiae* Nab2 Zn fingers 5–7 (ZnF567) induces formation of a heterotetramer containing two protein and two RNA chains, whereas ZnF567 alone is monomeric in solution. The crystal structure of ZnF567 bound to A_11_G RNA indicated that there are few direct interactions between the protein chains in the heterotetramer and that each RNA chain binding to two protein chains mediates the generation of a ZnF567 dimer. Dimerization of the Zn fingers generated by RNA binding provides a basis for understanding the function of Nab2 in both compacting and packaging mature transcripts for nuclear export as well as controlling 3΄ poly(A) tail length.

## MATERIALS AND METHODS

### Proteins and RNA

A fragment of *S. cerevisiae* Nab2 corresponding to ZnF567 (residues 409–485) and the corresponding F450A mutant were expressed in *Escherichia coli* and purified as described (18). Synthetic RNAs (A_12_ and A_11_G) were purchased from Integrated DNA Technologies (Belgium). The full-length mRNA open reading frame of the *S. cerevisiae GAL1* gene was produced by *in vitro* transcription. The gene was amplified by PCR and sub-cloned into a pUCIDT (amp) vector containing an upstream T7 promoter and a downstream Xho1 site for effective linearization of the vector. The run-off transcription reaction contained 0.04 mg/ml T7 RNA polymerase mixed with 100 ng/μl DNA template, 7.5 mM NTPs, 50 mM MgCl_2_, 1 mM spermidine and 80 mM Na–HEPES buffer, pH 7.5, and incubated at 37°C for 4 h. The RNA was purified by phenol/chloroform extraction at pH 4.5 followed by ethanol precipitation and was stored at −20°C.

### Biophysical determinations

Size exclusion chromatography-multiangle light scattering (SEC-MALS) employed a Superdex 200 10/30 column coupled to a Wyatt Heleos II 18 angle light scattering instrument coupled to a Wyatt Optilab rEX online refractive index detector (Wyatt Technology, Haverhill, UK) as described (21). Protein and RNA concentrations were determined from the excess differential refractive index (ΔRI), based on values of 0.193 for 1 mg/ml protein, 0.220 for 1 mg/ml RNA and 0.206 for 1 mg/ml protein–RNA complexes. Measurements were performed at 20°C in 50 mM Tris pH 8.5, 100 mM NaCl, 2 mM Mg acetate, 100 μM ZnCl_2_, 1 mM DTT using a flow rate of 0.5 ml/min. The molar mass was determined as described (21). Isothermal calorimetry (ITC) was performed using an iTC200 calorimeter (Malvern Instruments, Malvern, UK) in 50 mM Tris–HCl pH 8.5, 50 mM NaCl, 10 μM ZnCl_2_ and 1 mM DTT as described (18) by adding aliquots of 200 μM RNA to 30 μM *S. cerevisiae* ZnF567. Because Nab2 ZnF567 does not contain tryptophan it was difficult to determine its precise concentration and so when calculating binding constants the stoichiometry of each measurement was normalized to 1 based on the RNA concentration, which was determined from the absorption at 260 nm. Native agarose gel electrophoresis was used to examine the influence of Nab2 Zn fingers 5–7 binding on *GAL1* RNA that was stained with SYBR Green II. Similarly, the binding of *GAL1* RNA by Nab2 ZnF567 was monitored using native PAGE stained with Coomassie Brilliant Blue.

### Crystallography

Crystals were obtained using sitting drop vapor diffusion using 30% ethanol, 10% PEG 6000, 0.1 M Na acetate as precipitant. 200 nl of complex, formed by adding a 10% molar excess of A_11_G RNA to ZnF567, was mixed with 200 nl of well buffer and crystals appeared after several days. Crystals were cryoprotected in well buffer supplemented with 20% glycerol, vitrified by plunging into liquid nitrogen, and data collected at 100 K on beamine I04-1 of the Diamond Light Source using a wavelength of 0.9282 Å and a Dectris Pilatus 2M image plate detector. Data were processed and reduced using *XDS* (22) and *AIMLESS* (23). Initial phases were obtained using single anomalous dispersion (exploiting the anomalous signal of the Zn atoms present) using the AutoSol automated protocols in the *PHENIX* suite (24). Iterative cycles of refinement using *phenix.refine*, with local rebuilding in *COOT* (25) generated a final structure with *R*_work_/*R*_free_ = 13.1/15.2% and excellent stereochemistry (Table 2) with a MolProbity (26) score of 0.67 (100th percentile).

### Electron microscopy

Samples for electron microscopy were prepared using the GraFix procedure (27) in which *GAL1* RNA (100 nM) and Nab2 fingers 5–7 (5 μM; 50-fold molar excess) were mixed in 25 mM HEPES buffer, pH 8 and then separated on a 10–30% (w/v) sucrose density gradient in which 0.25% (v/v) of glutaraldehyde had been added to the 30% sucrose solution used to make the gradient. Samples were centrifuged in a Ti60 rotor (Beckman Coulter, Brea, CA, USA) for 18 h at 4°C and seven-drop fractions collected and examined following negative staining with 2% (w/v) uranyl acetate. Micrographs were recorded at 23 000× using Techni Spirit or T12 electron microscopes operated at 120 kV and equipped with Gatan Orius and Ultrascan 1000 CCD cameras, respectively.

## RESULTS

### A_11_G and A_12_ RNA induce dimerization of Nab2 Zn fingers 5–7

**Figure 2.**
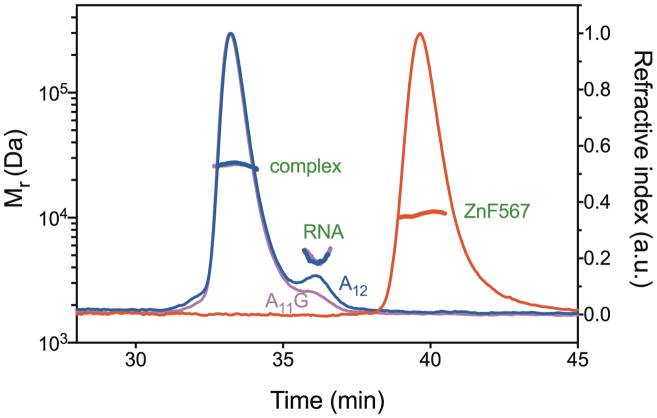
Multiangle light scattering data. Nab2 Zn fingers 567 (red) and the complexes formed with A_11_G (magenta) and A_12_ (blue) RNA that had an Mr consistent with the formation of a heterotetramer containing two protein and two RNA chains. A small excess of RNA was added to ensure complete complex formation. In contrast to the complexes formed with RNA that contained two protein chains, Nab2 Zn fingers 567 alone was monomeric.

ITC indicated that both A_12_ and A_11_G RNAs bound to Nab2 Zn fingers 5–7 (ZnF567) with an ∼1:1 stoichiometry and comparable affinity, although A_11_G RNA bound with a marginally higher affinity than A_12_ RNA (0.3 and 0.4 μM, respectively, Supplementary Figure S1). SEC-MALS indicated the Mr of protein alone was 10.2–10.7 kDa (theoretical: 9.264 kDa), whereas those for A_12_ RNA (theoretical: 3.950 kDa) and A_11_G RNA (theoretical: 3.966 kDa) were 4.8 and 4.6 kDa, respectively, consistent with their being monomeric in solution (Figure 2 and Table 1). However, although SEC-MALS indicated that the 1:1 complex formed between ZnF567 and A_11_G RNA was also monodisperse (Figure 2 and Table 1), its mass was 26.8 kDa (calculated Mr for two protein chains and two RNA chains is 26.778 kDa) and similarly the mass of the complex with A_12_ RNA was 26.4 kDa (calculated Mr for two protein chains and two RNA chains is 26.428 kDa), indicating that binding either A_11_G and A_12_RNA induced ZnF567 dimerization leading to the formation of a heterotetramer that contained two protein and two RNA chains.

**Table 1.** Size exclusion chromatography-multiangle light scattering (SEC-MALS) data

Sample	Mr (kDa)	
	Theoretical	Observed
Nab2 ZnF5-7	9.264	10.7
A_12_ RNA	3.950	4.8
A_11_G RNA	3.966	4.6
Nab2 ZnF5-7 + A_12_ RNA	13.214/26.428^a^	26.4
Nab2 ZnF5-7 + A_11_G RNA	13.230/26.460^a^	26.8
Nab2 ZnF5-7^F450A^ + A_12_ RNA	13.138/26.276^a^	14.4

^a^(1:1 complex)/(2:2 complex).

### Crystal structure of Zn fingers 5–7 bound to A_11_G RNA

**Figure 3.**
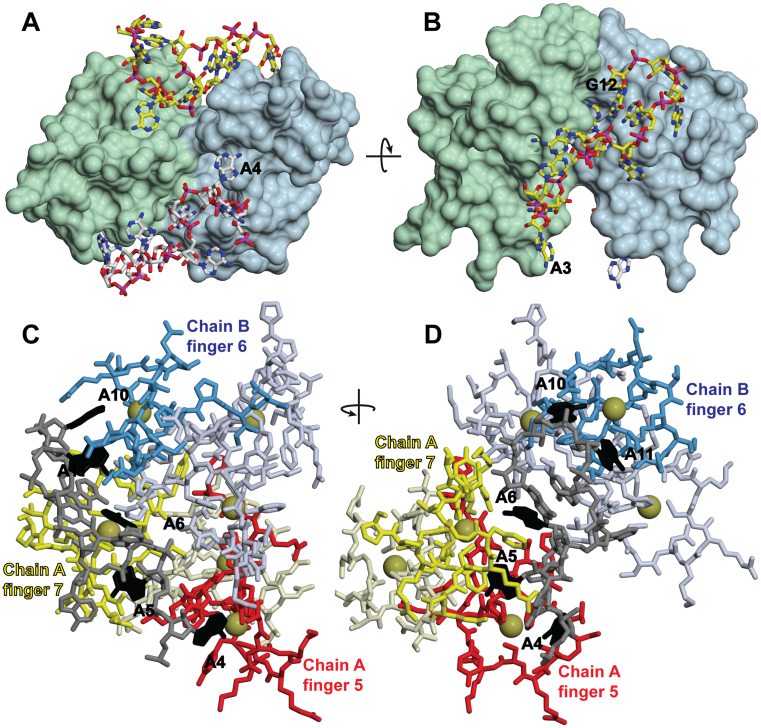
Crystal structure of the ZnF567:A_11_G complex. **(A, B)** Overview of a heterotetramer containing two protein and two RNA chains showing how the adenine purine rings are buried into cavities in the surface of the Zn fingers. The dimerization of the two Nab2 Zn finger protein chains is mediated by each RNA chain binding to both protein chains (shown here as green and light blue), thereby linking them together. (**C, D**) Each RNA chain (gray) was bound to fingers 5 (red) and 7 (yellow) of one protein chain and finger 6 (blue) of the other protein chain. The interacting adenines are black. A4 was bound to finger 5 of chain A; A5 and A6 to finger 7 of chain A; and A10 and A11 to finger 6 of chain B (shown schematically in Figure 4A). Zn atoms are represented by gold spheres.

Crystals with *P2_1_* symmetry that diffracted to 1.55 Å resolution were obtained for the complex of A_11_G RNA with ZnF567. Initial phases were obtained using the Zn anomalous signal and iterative cycles of refinement using PHENIX (24) and rebuilding in COOT (22) generated a model with an *R*-factor of 13.1% (*R*_free_ = 15.2%), excellent geometry (Table 2) and a MolProbity (26) score of 0.67 (100th percentile). The asymmetric unit contained two heterotetramers that each contained two RNA and two protein chains (Figure 3). The final model contained 3277 non-hydrogen atoms (average *B*-factor 29.22 Å^2^) and 685 water molecules (average *B*-factor 39.34 Å^2^). Residues 409–479 were clear in all chains with density corresponding to the N-terminal Gly-Ser from TEV cleavage and residues 480–481 sometimes present. Electron density corresponding to residues 482–485 (that were disordered in the solution structure—(18)) was not observed. For the RNA chains, density was not observed for A1 and that for A2 was weak, but the density from A3-G12 was easily interpretable. Each heterotetramer contained A4-G12 with A3 forming a crystal contact with an adjacent heterotetramer. Although in all four RNA chains the backbone conformation of A7 and A10 were flagged by Molprobity (26) as outliers, the collection of RNA backbone conformers currently available (28) is not thought to be complete and, moreover, RNA at binding interfaces show a high number of nucleotides with unusual conformations (29). The clear 2*F*_o_ − *F*_c_ density for these ribonucleotides after refinement (Supplementary Figure S2) was consistent with their geometry being appropriate.

**Table 2.** Crystallography data collection and refinement statistics

***Crystal parameters***	
Space group	*P2_1_*
Unit cell	
*a, b, c* (Å)	46.67, 85.04, 64.96
α, β, γ (°)	90, 99.07, 90
***Data collection***	
Wavelength (Å)	0.9282
Resolution range (Å)	46.08–1.55 (1.58–1.55)
Total reflections	461 396 (16 873)
Unique reflections	72 125 (3527)
Multiplicity	6.4 (4.8)
Completeness (%)	99.4 (98.9)
Mean *I*/σ(*I*)	20.8 (1.9)
Anomalous completeness (%)	97.9 (95.5)
Anomalous multiplicity	3.2 (2.5)
Wilson *B*-factor	18.1
*R*_pim_	0.028 (0.543)
***Refinement***	
*R*-factor/*R*_free_	0.131/0.152
Bonds RMS (Å)	0.008
Angles RMS (°)	0.94
Ramachandran favored (%)	100
Ramachandran outliers (%)	0
Rotomer outliers (%)	0
MolProbity score (percentile)	0.67 (100%)

Values for the highest-resolution shell are shown in parentheses. The *R*_free_ set contained 5% of the reflections.

**Figure 4.**
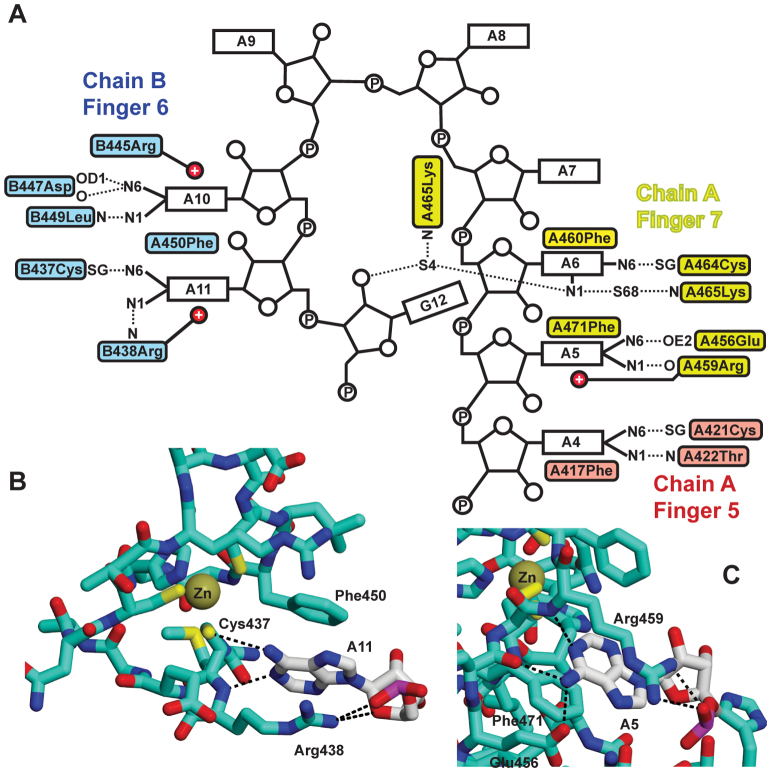
Interactions between Nab2 Zn fingers 5–7 and adenine bases. (**A**) Schematic illustration of the interactions between A_11_G RNA and Zn fingers 5–7. On one protein chain, Zn finger 5 (red) binds to base A4 while Zn finger 7 (yellow) binds bases A5 and A6, whereas in the second, protein chain in the dimer Zn finger 6 (blue) binds bases A10 and A11. In addition to hydrophobic interactions with an aromatic side chain and putative π interactions with a basic side chain, the specificity of the interaction involves H-bonds formed by the adenine N6 to either a SG of the finger or an appropriately positioned acidic side chain. Two different types of interaction between the purine ring and the protein mediate specific recognition of adenine. In one interaction, illustrated by A11 (**B**), the purine stacks against an aromatic side chain (Phe450) and forms π interactions with a basic side chain (Arg438) together with N6 forming a H-bond to the SG of one of the cysteines (Cys437) that is coordinated to Zn. In the second interaction (**C**), illustrated by A5, the purine ring is stacked against Phe450 and Leu449 on one side and forms π interactions with Arg445 on the other, as well as forming H-bonds between its N6 and the carbonyl and OD1 of Asp447 as well as between N1 and the amide of Leu449.

The heterotetramers present in the crystal were consistent with the Mr of 26.8 kDa observed in solution (Table 1). Within a heterotetrameter, each of the two RNA chains bound to both protein chains through interactions that were dominated by the purine rings of five of the bases being buried in surface cavities (Figures 3 and 4 and Supplemental Movies 1 and 2). In each protein chain, finger 5 bound to A4 and finger 7 bound to A5 and A6 of one RNA chain, whereas finger 6 bound to A10 and A11 of the other RNA chain (Figure 4). The 3΄ G12 did not interact directly with either protein chain, but appeared to contribute to the looping of the second half of the RNA chain that positioned A10 and A11 to facilitate interaction with finger 6. Previous work (19) has shown that the key interacting Nab2 residues (Phe417, Arg438, Phe450, Arg459, Phe460 and Phe471) are strongly conserved across different species. There were only trivial differences between the protein chains in the asymmetric unit (rmsd for all atoms of residues 411–477 was 0.90–1.08 Å) and the backbone of ZnF567 followed closely that of the NMR structure (18) of the apo-form (Supplementary Figure S3—the Cα RMSD between chain A and the average of the NMR ensemble was 1.39Å), indicating that RNA binding had not generated a substantial conformational change. ZnF567 dimerization was mediated primarily by each RNA chain binding to two protein chains (Figures 3 and 4) in an arrangement that was facilitated by the specific spatial arrangement of the three fingers so that the polyribonucleotide bound to fingers 5 and 7 of one chain and finger 6 of the other. Consistent with this hypothesis, mutation of Phe450, that interacts with both adenines bound to finger 6, impaired dimer formation and the Mr observed by SEC-MALS of the complex formed with A_12_ RNA and ZnF567^F450A^ was 14.4 kDa (Table 1), consistent with a 1:1 protein:RNA complex (Mr calculated for 1:1 complex is 13.138 and 26.276 for a 2:2 complex).

The adenine purines were recognized in two ways by *S. cerevisiae* ZnF567 (Figure 4). With A4, A6 and A11, a H-bond between Adenine N6 and the SG of a cysteine in the finger was supplemented by the purine stacking on one side against an aromatic side chain while its other face formed putative π interactions with a positively-charged residue (Figure 4B). Although the second binding geometry (Figure 4C) also stacked the purine of A5 and A10 against an aromatic side chain and formed π interactions, N6 instead formed a H-bond with a main chain carbonyl. In both cases, the H-bonds required the N6 hydrogens and so could not be formed by guanine O6, accounting for the specific recognition of adenosine by the fingers. No direct interactions were observed between the bases of A2–3 or A7–9 and the ZnF567 chains. In addition to the interactions with the adenine bases, binding was augmented by non-specific interactions with the RNA backbone including neutralization of the phosphates by basic residues on ZnF567 (e.g. between A11 OP1and Arg438, or between A7 OP2 and Arg459). G12 did not participate in any direct interactions with the protein except for though a water-mediated H-bond to Lys465, but may have contributed to the overall conformation of the RNA backbone though formation of a putative H-bond to A8 OP2.

Earlier attempts to identify poly(A) binding residues in ZnF567 using NMR (18) were frustrated because adding RNA containing more than three nucleotides resulted in the spectrum becoming too broad. These difficulties can now be understood in terms the considerable increase in size of the complex that resulted from poly(A) generating heterotetramers. Previous studies (18) showed considerable (at least 10-fold) reductions in the affinity of ZnF567 for A_8_ RNA coupled with an increase in poly(A) tail length *in vivo* when many of the key residues identified in the crystal structure (Lys416, Phe417, Arg438, Phe450, Arg459 and Phe460) were mutated, but did not identify the importance of Cys421, Cys437, Cys464 (mutation of which would have impacted on the structure of the finger), Asp447 and Glu456, or the main chain interactions involving Thr422, Arg438, Asp447, Leu449 and Arg459. The interactions of the adenine bases with ZnF567 were generally similar to those observed in crystals in which A_8_ RNA was bound to *C. thermophilum* Nab2 fingers 3–5 that have sequence similarities to Nab2 ZnF567 (19). With the *C. thermophilium* crystals, the A_8_ RNA employed was too short to generate 2:2 heterotetramers and instead these crystals showed three short RNA chains that were bound to different pairs of Nab2 chains to generate a network like arrangement rather than the heterotetramers observed with *S. cerevisiae* Nab2 ZnF567. The molecular recognition of adenosine by ZnF567 differed considerably from that observed for interactions between adenines and other RNA-binding proteins. Although the way in which adenines stacked against an aromatic residue and formed putative π interactions with a basic residue in Nab2 has been frequently observed when single-stranded RNAs bind to proteins (30–32), the pattern of H-bonds that generated the specificity of the interaction (Figure 4) was quite different to those observed previously, especially in the involvement of a SG from one of the Zn-binding cysteines in each finger. Thus, when TIS11d binds UAUU RNA, adenine N6 and N7 form H-bonds to main-chain carbonyls or amides and do not interact with the SGs from the Zn-coordinating cysteines (33), whereas the adenines in the Unkempt and MBNL1 RNA motifs form a network of H-bonds primarily with the protein main chain (34,35).

In summary, the crystal structure of the complex formed between Nab2 Zn fingers 5–7 and A_11_G RNA demonstrated how reciprocal interactions between A-rich sequences have the capacity to generate Nab2 dimers in a way that does not depend on interactions between the proteins themselves but depends critically on the unique spatial arrangement of the Zn fingers.

### Nab2 Zn fingers 5–7 compact GAL1 mRNA

**Figure 5.**
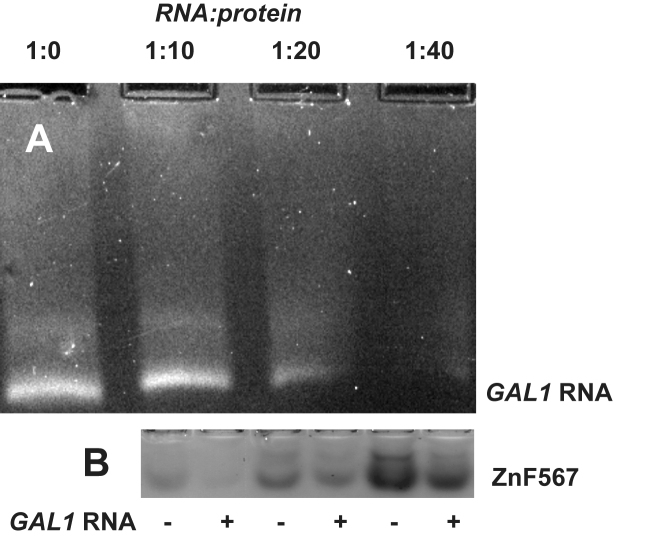
Binding of Nab2 ZnF567 to *GAL1* RNA. (**A**) Adding progressively larger amounts of ZnF567 depleted the amount of *GAL1* RNA that migrated to the positive electrode in native agarose gels. (**B**) Similarly, in native PAGE gels the Nab2 protein was depleted by *GAL1* RNA. At a 10:1 ratio most of the protein was depleted and there was substantial depletion even when it was added at a 40:1 ratio, consistent with each RNA binding multiple copies of the protein.

Because Nab2 has been found to bind to the coding region of transcripts in addition to the poly(A) tails (8,9,14), we investigated whether ZnF567 might also be able to bind to different regions of a transcript and, by generating complexes analogous to the heterotetramers produced by A_11_G RNA, might link them together and thereby facilitate compaction. As a proof-of concept, a transcript generated *in vitro* corresponding to the coding sequence of *GAL1* was used as a model system and binding was assessed using gel electrophoresis, exploiting the difference in charge between the RNA (negative) and the protein (positive). Thus, when mixed with ZnF567 the density of the *GAL1* RNA band decreased, coupled with formation of a heterogeneous mixture of different species species that migrated more slowly towards the positive electrode (Figure 5A), consistent with ZnF567 binding to regions of the *GAL1* RNA coding sequence. The heterogeniety observed probably resulted from both different numbers of ZnF567 being bound to individual transcripts and also from their binding in different ways, consistent with the appearance of these complexes observed by electron microscopy (see below). A similar depletion of the ZnF567 protein band on SDS PAGE was observed on the addition of increasing amounts of *GAL1* transcript (Figure 5B).

**Figure 6.**
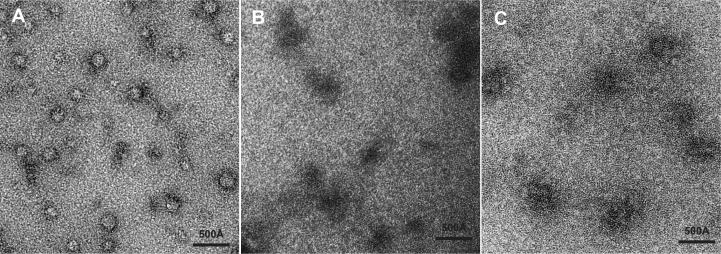
Compaction of GAL1 RNA by Nab2 ZnF567. (**A**) Electron micrograph of complexes formed between *GAL1* RNA and ZnF567 negatively stained with uranyl acetate following GraFix fixation. Fields contained roughly spherical stain-excluding particles of diameter of the order of 120 Å, consistent with the RNA becoming compacted following binding of the protein. (**B**) By contrast, rather than excluding uranyl acetate, in the absence of ZnF567 *GAL1* RNA bound the stain and so appeared dark as is typically seen with nucleic acids (37). (**C**) When ZnF567^F450A^ was bound to *GAL1* RNA, the particles were less compact than those obtained with the wild-type protein.

Electron microscopy indicated that the binding of ZnF567 to *GAL1* RNA generated compacted complexes. Thus, when *GAL1* RNA and ZnF567 were mixed and then fractionated using the Grafix procedure (27), micrographs of material negatively stained with uranyl acetate (Figure 6A) showed the presence of roughly spherical particles comparable to the size of bacterial ribosome 30S particles and which had diameters of the order of 120 Å (mean = 118 Å; SD = 24 Å, *n* = 53), consistent with ZnF567 alone mediating some compaction the *GAL1* transcript. Although there was considerable heterogeneity both in the size and shape of these particles, this was probably a result of the Nab2 Zn fingers linking different regions of the RNA chain in different particles and perhaps, at times, also crosslinking two transcripts together to form a larger particle. RNA alone binds uranyl acetate strongly and so appears dark in electron micrographs (41) so that *GAL1* transcripts alone did not exclude the negative stain and, so unlike the protein:RNA complexes, appeared as dark amorphous areas in electron micrographs (Figure 6B). The particles in electron micrographs of *GAL1* transcripts mixed with ZnF567^F450A^ (Figure 6C) were similar in size to the RNA alone and were more open than the particles seen with wild-type ZnF567, consistent with impaired dimerization producing reduced compaction.

## DISCUSSION

Complementary information from both solution studies (Table 1) and crystallography (Figures 3 and 4) identified that binding of either A_12_ or A_11_G RNA to *S. cerevisiae* ZnF567 generated a heterotetramer containing two protein and two RNA chains. This complex was generated by each RNA chain binding to both protein chains rather than ZnF567 dimers being generated by direct interaction between the protein chains and this precluded the generation of engineered mutants that could disrupt dimeriztion without targeting RNA binding. This novel dimerization was generated by the unique spatial arrangement of the Zn fingers that impaired a single RNA chain binding to all three fingers in each protein chain. In addition to H-bonds involving N6, adenine bases interacted with appropriately positioned hydrophobic side chains on one side and often formed putative π bonds to a basic residue on the other side (Figures 3 and 4). Although detailed structural information is not yet available for the interactions involving Zn fingers 1–4, they have similar folds to fingers 5–7 and are grouped in two pairs (1+2 and 3+4) and several show preferred binding to poly(A) RNA (17), albeit more weakly than ZnF567. It is probable that when the Nab2 chains have been dimerized by fingers 5–7 binding to RNA, the remaining fingers bind to adjacent A-rich sequences. The generation of heterotetramers containing ZnF567 dimers provides a basis for understanding the function of Nab2 in both regulation of poly(A) tail length and in the packaging of transcripts to generate compact export-competent mRNPs.

**Figure 7.**
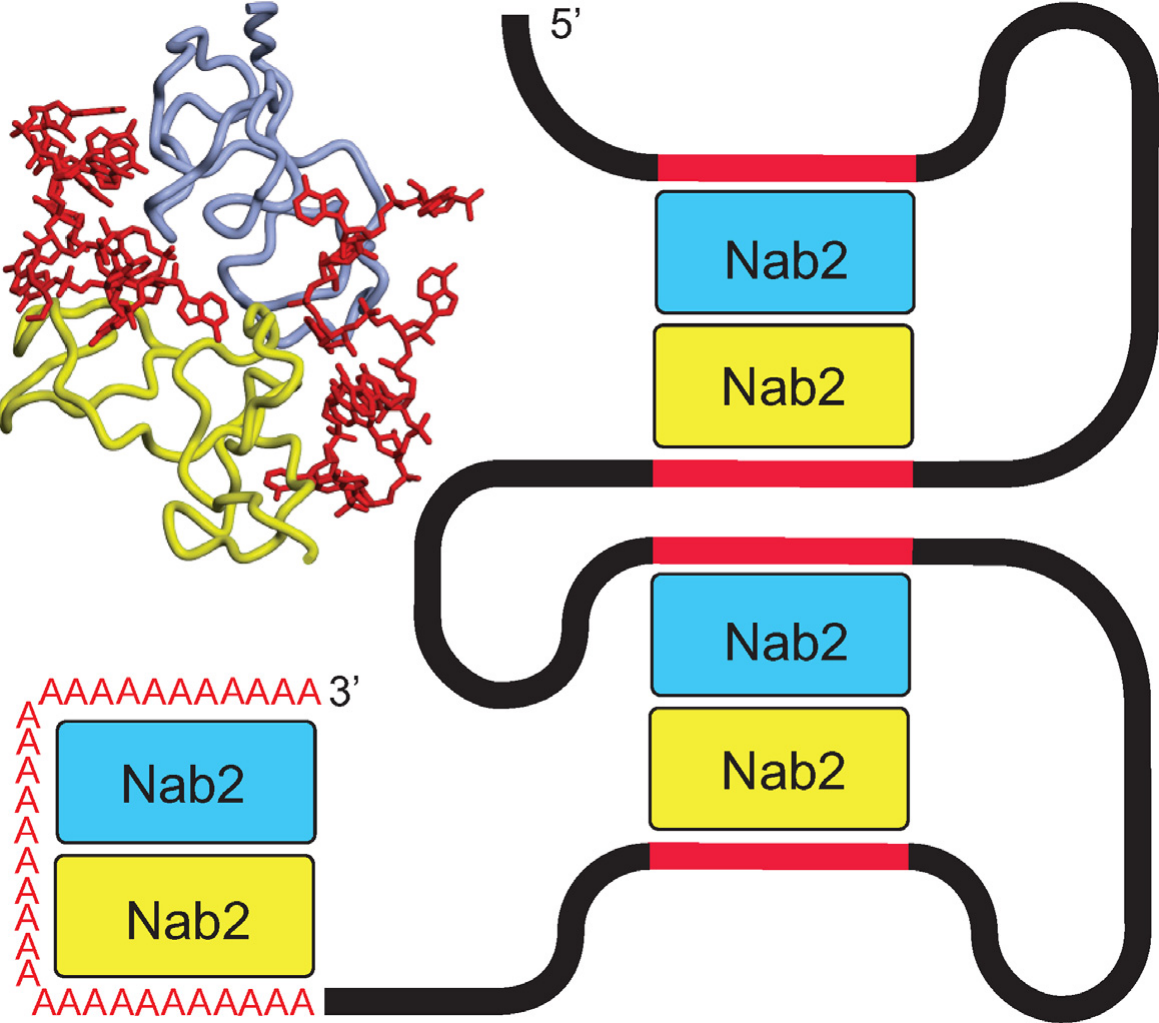
Schematic illustration of Nab2 function in polyadenylation and compaction. A poly(A) tail length of ∼60 nt (red As) could be generated by RNA-binding mediated Nab2 dimerization with each Nab2 binding ∼30 nt. Similarly, Nab2 dimers generated by binding A-rich regions of the coding sequence (red sections) would link different parts of the transcript resulting in compaction.

The dimerization of Nab2 induced by poly(A) RNA binding provides a rationale for the function of Nab2 in controlling poly(A) tail length (Figure 7). Polyadenylation is generally considered to be the last major processing step in the nuclear phase of the gene expression pathway and, in mature *S. cerevisiae* mRNAs, the poly(A) tails are typically ∼60 nt long. However, a large number of Nab2 mutants show increased poly(A) tail length *in vivo* (reviewed by (7)). Viphakone *et al.* (13) showed that individual Nab2 chains bound ∼30 As and proposed that binding of two Nab2 molecules to the growing poly(A) tail resulted in termination. Therefore the generation of a heterotetramer in which two Nab2 chains each bind ∼30 adenines would generate a length of ∼60 nucleotides and would account for why only two Nab2 chains, rather than a larger number, are involved in binding poly(A) tails. Mutations in the Nab2 Zn fingers that impaired heterotetramer formation, either by disrupting its geometry or reducing the affinity of a finger for adenine, would be expected to result in less efficient termination of polyadenylation and so account for the increased poly(A) tail length observed *in vivo* (18,19). The hypothesis that Nab2 dimerization contributes to poly(A) tail length determination was supported by the results obtained with the F450A mutant that impaired ZnF567 dimerization (Figure 2) and which previous work has shown results in a considerable increase of poly(A) tail length *in vivo* (18). Although further work will be necessary to define precisely how the Nab2 dimerization mediated by RNA binding limits poly(A) tail length, in principle this could be achieved either by its facilitating the termination of polyadenylation or, alternatively, limiting any trimming of the poly(A) tail by the exosome to the region not bound to Nab2.

Dimerization of Nab2 mediated by its binding to two separate A-rich sequence motifs in a transcript would be an effective way to link two regions of a RNA chain together to facilitate compaction (Figure 6). Batisse *et al.* (8) showed that the RNA chain in most export-competent *S. cerevisiae* mRNPs is compacted by at least a factor of ten to facilitate passage through NPCs. Although a host of different proteins bind to transcripts as they progress through the nuclear processing stages of the gene expression pathway, only a small number appear to accompany mature transcripts to the cytoplasm (1). Although composition probably varies between transcripts, several mRNA binding proteins, including cap-binding proteins, the export factor, Mex67:Mtr2, the SR protein, Npl3, and Nab2, are thought to be present on most, if not all, export-competent mRNPs in *S. cerevisiae* (5), albeit metazoan mRNPs appear to also have some additional RNA-binding proteins, such as those of the exon-junction complex. Although Nab2 binds to 3΄ poly(A) tails of mature mRNAs, crosslinking and other studies have demonstrated that it also binds along the entire coding sequence (9,14), which would be required for it to function in compaction. Moreover, the crystal structure of ZnF567 bound to A_11_G indicates that the interaction only requires adenines in selected positions in the motif (here bases 4,5,6,10 and 11) and so only an A-rich region, possibly terminating in G, as suggested by SELEX (14) and computational studies (16), would be required rather than a full A_11_G motif. Moreover, results obtained with other RNA binding proteins, such as the Pumilio family (36) or Unkempt (35), indicate that there can be a level of plasticity in RNA motifs and frequently an additional base can be inserted between key interacting bases. The results obtained by gel electrophoresis (Figure 5) and electron microscopy (Figure 6) on ZnF567 support the hypothesis that Nab2 Zn fingers contribute to the compaction of transcripts to facilitate export to the cytoplasm through NPCs. Although the particles generated *in vitro* were heterogeneous, probably as a result of different regions being linked within individual transcripts (that probably also contained a range of different secondary and tertiary conformations—see (38)) and also crosslinks sometimes being formed between rather than within transcripts, these results illustrate that binding Zn fingers 5–7 had the potential to generate relatively compact complexes with *GAL1* RNA. *In vivo* this heterogeneity would probably be reduced because Nab2 molecules would probably be added sequentially as transcription proceeded and crosslinks between transcripts would be less likely. Of course the results obtained with ZnF567 should only be considered as a proof of principle. In living cells full-length Nab2 together other factors will also contribute to compaction to generate the approximately cylindrical mRNPs observed by Batisse *et al.* (8). Although further work will be required to define precisely how Nab2 contributes to compaction, the electrophoresis and electron microscopy data indicate that the generation of structures analogous to the heterotetramers observed with A_11_G offers a structural basis for this function. Moreover, once mRNPs have reached the cytoplasm, it is necessary to reverse the compaction to facilitate translation and this could be readily achieved by the removal of Nab2 from mRNPs by the DEAD-box helicase Dbp5 at the cytoplasmic face of NPCs (39,40).

In summary, biophysical and structural methods demonstrate that binding A_11_G RNA induces dimerization of ZnF567 that is mediated by each RNA chain binding to two protein chains. The dimerization of Nab2 induced by RNA binding provides a basis for understanding its function in both poly(A) tail length regulation and in the compaction of mature transcripts to facilitate nuclear export.

## ACCESSION NUMBER

Coordinates and structure factors for the complex of Nab2 Zn fingers 5–7 with A_11_G RNA have been deposited with the protein data bank with accession code 5L2L.

## Supplementary Material

Supplementary Data
