# Supplementary material for: Structural basis for the dimerization of Nab2 generated by RNA binding provides insight into its contribution to both poly(A) tail length determination and transcript compaction in Saccharomyces cerevisiae
Source: Nucleic Acids Res. 2016 Dec 8;45(3):1529–38. doi: 10.1093/nar/gkw1224 (PMC5388407; doi:10.1093/nar/gkw1224)
Supplement: Supplementary Data [file gkw1224_Supp.zip › nar-02418-a-2016-File009.pdf]

Supplementary Information

Structural basis for the dimerization of Nab2 generated by RNA provides insight into its contribution to both poly(A) tail length determination and transcript compaction in *Saccharomyces cerevisiae*.

Shintaro Aibara, James M. B. Gordon, Anja S. Riesterer, Stephen H. McLaughlin and Murray Stewart

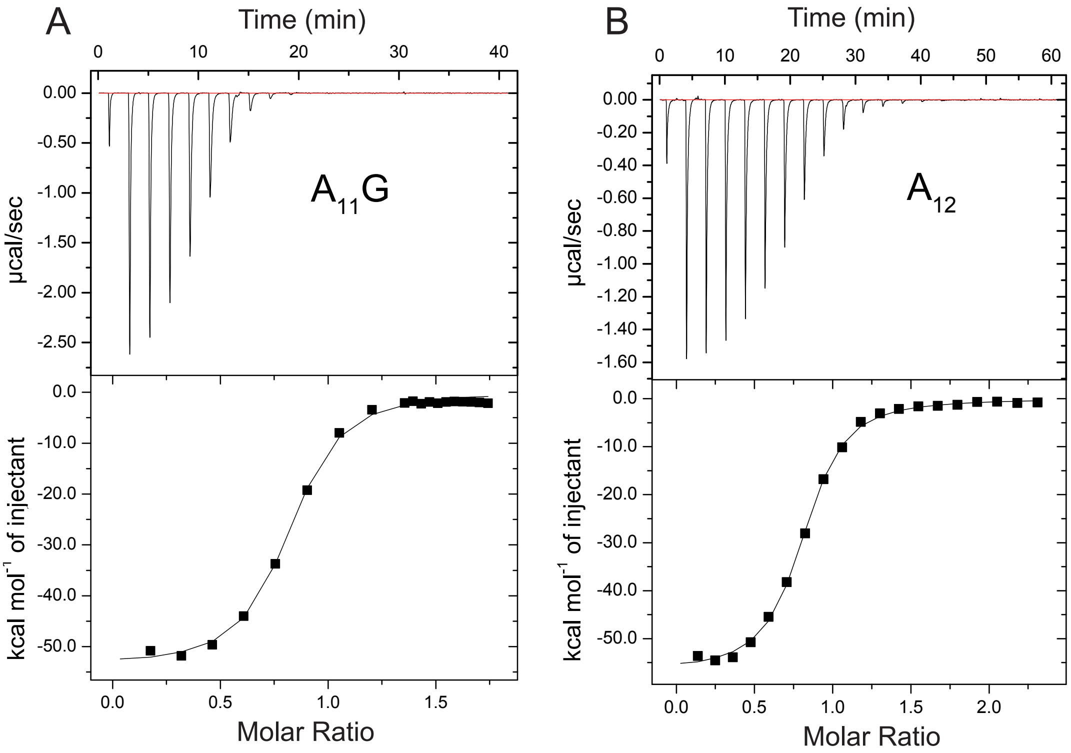

Supplementary Figure S1. ITC and MALs data for the interaction between Zn fingers 5-7 and either A_{12} (A) or $A_{11}G$ (B) RNA indicating 1:1 binding stoichiometry and formation of heterotetramers in solution. The binding of both ribonucleotides to fingers 5-7 showed similar thermodynamic properties with a favourable enthalphy change (ΔH -53.4 kcal/mol and -57 kcal/mol, respectively) together with an unfavourable entropy change (-TΔS 44.7 kcal/mol and 48.8 kcal/mol, respectively), which would be consistent with the interaction exhibiting enthalphy/entropy compensation (reviewed by Chodera and Mobley, 2013). Because Nab2 ZnF567 does not contain tryptophan it was difficult to determine its precise concentration and so when calculating binding constants the stoichiometry of each measurement was normalized to 1 based on the RNA concentration, which was determined from the absorption at 260 nm.

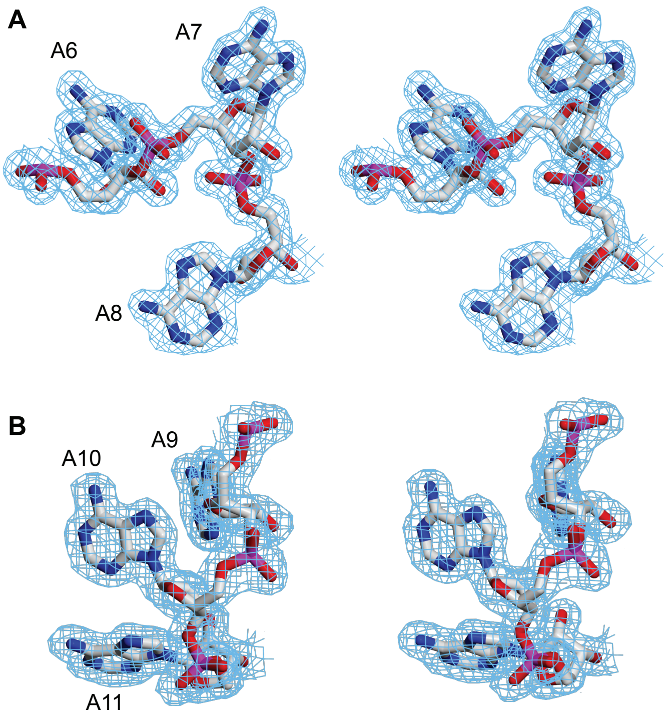

Supplementary Figure S2. Stereo views of the 2Fo-Fc electron density map surrounding adenines A7 (A) and A10 (B) consistent with these bases having a non-cannonical conformation.

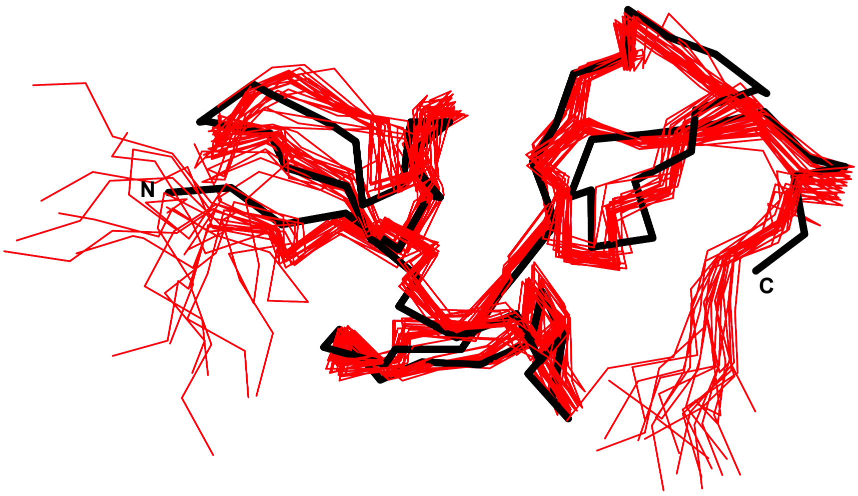

Supplementary Figure S3 . Superposition of the backbones of the Nab2 Zn finger 5-7 chain bound to $A_{11}G$ RNA (black) on the ensemble of solution structure of the apo-protein (red) indicating that a substantial conformational change did not accompany RNA binding. Because the NMR structure is represented as an ensemble, a Cα RMSD was calculated between chain A and the average of the NMR ensemble. This RMSD was 1.39Å, consistent with little structural change in ZnF567 being introduced by RNA binding.

Supplementary Movies

Movie 1. Illustration of how the two RNA chains (red) link the two different Nab2 chains (shown as Cα worm traces in yellow and blue) in the ZnF567 heterotetramer. Zn atoms are shown as silver spheres.

Movie 2. Illustration of how the two RNA chains (coded with standard atom colours) link the two different Nab2 chains (shown as spheres in green and blue) in the ZnF567 heterotetramer. The purine rings of A4, A5, A6, A10 and A11 are buried in surface cavities in the Zn fingers.

Supplementary References

Chodera, J.D. and Mobley, D.L. (2013). Entropy-enythalpy compensation: role and ramifications in biomolecular ligand recognition and design. *Ann. Rev. Biophys.* **42**, 121-142.
